# Aberrant expression of Arpin in human breast cancer and its clinical significance

**DOI:** 10.1111/jcmm.12740

**Published:** 2015-12-09

**Authors:** Xiangping Liu, Bin Zhao, Haibo Wang, Yu Wang, Mengdi Niu, Ming Sun, Yang Zhao, Ruyong Yao, Zhiqiang Qu

**Affiliations:** ^1^ Medical Research CenterThe Affiliated Hospital of Qingdao UniversityQingdaoChina; ^2^ Department of UltrasonographyQilu HospitalShandong UniversityQingdaoChina; ^3^ Center of Diagnosis and Treatment of Breast DiseaseThe Affiliated Hospital of Qingdao UniversityQingdaoChina

**Keywords:** arpin, breast cancer, prognosis

## Abstract

Arpin (Arp2/3 complex inhibitor), a novel protein found in 2013, plays a pivotal role in cell motility and migration. However, the precise role of Arpin in cancer is unclear. This study investigated the expression of Arpin in breast cancer and evaluated its correlation with the characteristics of clinical pathology and prognosis of breast cancer patients. Immunohistochemistry (IHC) for Arpin protein was performed on formalin‐fixed, paraffin‐embedded 176 breast cancer tissues and 43 normal breast tissues while qRT‐PCR for Arpin mRNA with 104 paired tumour and paratumoural tissues from breast cancer patients respectively. The association of Arpin expression with clinical pathological features and survival was assessed in a retrospective cohort analysis of patients. The results showed that the expression of Arpin protein in cancer tissues was lower compared to that in normal breast and the expression of Arpin mRNA was also lower in cancer tissues than that in the matched paratumoural tissues. Among the 176 breast cancer patients, the lower expression of Arpin was significantly associated with advanced tumour, nodes and metastasis system stage, and the reduced Arpin expression was strongly associated with axillary lymph node metastasis using univariate and multivariate logistic regression analysis [odds ratio: 3.242; 95% confidence interval (CI): 1.526, 6.888; *P* < 0.05]. Furthermore, Arpin expression was an independent risk factor for recurrence‐free survival (HR: 0.373; 95% CI: 0.171, 0.813; *P* < 0.05). As Arpin expression was first examined in human breast cancer tissues with qRT‐PCR and IHC, our results suggest that Arpin downregulation may contribute to the initiation and development of breast cancer metastasis. Therefore, as a potential predictive marker, Arpin deserves future studies.

## Introduction

Breast cancer is the most common malignancy in females with an increasing annual incidence and it is usually accompanied by local invasion and distant metastasis, which are the main causative factors for cancer‐related death 1, 2. Clinical outcome of breast cancer patients is widely variable, due to the genetic alterations of breast cancer which are increasingly recognized as critical determinants of tumour initiation, development, local invasion and distal metastasis 3, 4. More and more studies have shown that the metastatic capability of cancer is empowered by the molecular changes which may exist in the early stage of tumourigenesis 5. Therefore, elucidation of these factors and the pattern of their expression may help to better understand the progression of breast cancer, and help to predict the clinical outcome of breast cancer patients. Furthermore, the capacity to catalogue the genetic alterations associated with breast cancer may facilitate advances in its treatment.

Lamellipodia is a major tool for cancer cells to explore their extra‐environment and to form new adhesion contacts with the extracellular matrix for motility and spreading. The common process of the formation of lamellipodia is driven by spatially and temporally regulated actin polymerization at the leading edge 6. And actin polymerization is regulated by a very important molecule—Arp2/3 complex—which is considered to be a key regulator of cell motility 7, and was reported to be involved in the development and migration of some cancers such as pancreatic cancer, gastric cancer, colorectal cancer, breast cancer and so on 8, 9, 10, 11. Lamellipodia drives the motion of the cell through changing actin cytoskeletons such as assembling new actin or retracting the protrusive actin structure. Arp2/3 complex is a major controller of actin assembly and initiator of new actin filament in lamellipodia 12. By binding actin filaments to the side of an existing filament and initiating branch formation, Arp2/3 complex accomplishes its mission of nucleating actin filaments 13.

Arpin is a newly found Arp2/3 complex inhibitor reported in Nature, 2013. Arpin is encoded by the gene C15ORF38 and its protein is encoded by a single gene containing 220 residues and is structured with the exception of its highly mobile C‐terminal end, which contains the putative binding site of Arp2/3 complex 14, which has been reported to be closely related to the development and migration of cancer cells for its key role in the filopodia initiation 15, 16. Arpin and its acidic motif could compete with nucleation promoting factors for Arp2/3 binding and thereby inhibit the activation of the Arp2/3 complex 14, 17.

Furthermore, Arpin is involved in the regulation of the motility process in a variety of cells, including *Dictyostelium discoideum* amoeba, keratocytes and human breast cancer cell line MDA‐MB‐231 14. Arpin‐depleted MDA‐MB‐231 cells can invade a larger territory than the control cells that contain Arpin in two or three dimensions 14. However, little is known about whether the expression of Arpin is altered in human breast cancer tissues and how this expression is correlated with the clinicopathological characteristics and prognosis of the patients.

In this study, we employed qRT‐PCR and immunohistochemistry (IHC) to examine the expressions of Arpin gene and protein in breast cancer tissues, and assessed its correlation with clinicopathological and prognostic variables of patients with breast cancer. We observed a markedly low expression level of Arpin in cancer tissues of patients and postulated that low expression level of Arpin may serve as a risk factor that is predictive of poor prognosis in patients with breast cancer.

## Materials and methods

### Patients and sample collection

Formalin‐fixed, paraffin‐embedded specimens (43 normal breast tissues *versus* 176 breast cancer tissues) for IHC assay were collected from breast patients who underwent surgical resection between January 2009 and June 2009 at the Affiliated Hospital of Qingdao University. Also, 104 paired tumour tissue and paratumour tissue samples (3–5 cm from the border of the tumour) for qRT‐PCR assay came from curative resection specimens of breast cancer patients (January–July 2014) at the same hospital. Samples were histologically diagnosed for primary adenocarcinoma or normal tissue by haematoxylin and eosin staining. None of the patients had received radiation therapy or chemotherapy before the surgery. The following data were retrieved and used as covariates in multivariate analyses: age, tumour size, lymph node status, expression of oestrogen receptor (OR), progesterone receptor (PR), human epidermal growth factor 2 (HER2), Ki‐67 (cut‐off value: 20%), tumour, nodes, metastasis system (TNM) stage and histological type. All patients were followed up periodically for survival analysis until recurrence or the study closing date (March 2014). The median follow‐up was 61.0 months (range 11.9–63.0 months). Follow‐ups were performed through home visits and telephone calls every 3 months. Recurrence‐free survival (RFS) was defined as the time from the primary operation date to the date of recurrence 18. All tumours were graded and histologically classified according to pathological standards by two experienced breast cancer pathologists who had no prior knowledge of the clinical features and outcomes of the patients. The study was approved by our Institutional Ethical Committee and written consent for using the samples for research purposes was obtained from all patients before surgery.

### Characteristics of patients

The following data about the patients were retrieved from the archives of our hospital. The median age (minimum, maximum) of all 280 cancer patients whose tissues were used for the IHC (176 cancer patients) and qRT‐PCR (104 cancer patients) assays was 51 years old. The TNM stages of specimens for IHC assay were 72 (40.9%) in TNM stage I, 52 (29.5%) in stage II and 52 (29.5%) in stage III respectively. And 14 (8.0%), 68 (38.6%) and 94 (53.4%) cases were assessed as G1, G2 and G3 according to histological grade. The TNM stages of specimens for qRT‐PCR assays were 34 (32.7%) in TNM stage I, 32 (30.8%) in stage II and 38 (36.5%) in stage III respectively. And 12 (11.5%), 48 (46.2%) and 44 (42.3%) cases were assessed as G1, G2 and G3 according to histological grade. The 43 normal breast tissues were from benign patients. The characteristics of the patients are shown in Tables 1 and 2.

**Table 1:** Association analysis of IHC staining for Arpin *versus* clinicopathological factors of breast cancer

Variable	Number of patients (*n* = 176)	Arpin expression		*P*‐value
		High (*n* = 56)	Low (*n* = 120)	
Age				
>50	79	26	53	0.779
<50	97	30	67	
Lymph node metastasis				
Yes	88	16	72	<0.001
No	88	40	48	
TNM stage				
I	72	32	40	0.003
II	52	16	36	
III	52	8	44	
Grade				
1	14	2	12	0.065
2	68	28	40	
3	94	26	68	
OR				
Positive	122	34	88	0.091
Negative	54	22	32	
PR				
Positive	132	36	96	0.025
Negative	44	20	24	
Her‐2				
Positive	26	6	20	0.3
Negative	150	50	100	
Ki‐67				
Positive	66	20	46	0.738
Negative	110	36	74	
Diameter				
≥2	52	12	40	0.107
<2	124	44	80	

Ki‐67 ≤ 20%: Negative.

Ki‐67 > 20%: Positive.

TNM: tumour, nodes, metastasis system; OR: oestrogen receptor; PR: progesterone receptor.

**Table 2:** Clinical characters of the enrolled cancer patients for qRT‐PCR

Variable	Number of cancer patients (*n* = 104)
Age	
>50	60
≤50	44
Lymph node metastasis	
Yes	64
No	40
TNM stage	
I	34
II	32
III	38
Grade	
1	14
2	46
3	44
OR	
Positive	52
Negative	52
PR	
Positive	36
Negative	68
Her‐2	
Positive	22
Negative	82
Ki‐67	
Positive	28
Negative	76
Diameter	
≥2	50
<2	54

Ki‐67 ≤ 20%: Negative.

Ki‐67 > 20%: Positive.

TNM: tumour, nodes, metastasis system; OR: oestrogen receptor; PR: progesterone receptor.

### Immunohistochemistry

Arpin IHC staining was performed on 4 μm‐thick sections of 10% formalin‐fixed, paraffin‐embedded tissue. Briefly, sections were boiled in a pressure cooker for 3 min. and placed in 3% hydrogen peroxide (H_2_O_2_) for 10 min. to inhibit endogenous peroxide activity. The sections were then washed with buffer and incubated with goat polyclonal antibody specific to Arpin (sc‐242049; Santa Cruz Biotechnology Inc., Bergheimer, Heidelberg, Germany, final dilution 1:25) at 4°C for 24 hrs, followed again by a buffer wash thrice. Biotinylated anti‐goat immunoglobulin was used as the second antibody. Subsequently, the sections were incubated with avidin–biotin‐conjugated peroxidase. Finally, the sections were washed and stained with 3,3‐diaminobenzidine tetrahydrochloride and hydrogen peroxide, and a brown pigment was obtained after counterstaining with haematoxylin.

### Immunohistochemical analysis and scoring

The tumour cells in which the cytoplasm was stained dark brown under light microscopy were considered positive. For the quantification of Arpin expression, both the staining intensity and the percentage of stained cells were evaluated. Cells without staining were scored as 0 points, weak staining intensity, 1 point, moderate staining intensity, 2 points, and strong staining intensity, 3 points. The percentage of stained tumour cells 0% was 0 points, less than 25%, 1 point, 25–50%, 2 points, or more than 50%, 3 points. The final score for Arpin expression was the sum of the above two kinds of scores. A staining score from 0 to 3 points was considered as low expression and a score more than 3 points was considered as high expression 19.

### Quantitative RT‐PCR

Primer design and synthesis Primer Express software (Applied Biosystems, Foster, CA, USA) was used to design primers specific to the amplification of Arpin gene by qRT‐PCR. Thereafter, the basic local alignment search tool was used to determine their specificity. A read‐through C15ORF38‐AP3S2 gene (NM_001199058.1) is easily confusing with Arpin gene (NM_182616.3), for it contains a majority of Arpin. So primers only targeting the Arpin gene were designed at the downstream of stop codon of Arpin, excluding the disturbance of the C15ORF38‐AP3S2 gene. The primers for the human Arpin gene were synthesized as follows: sense 5′‐AGACAACATCATGGCCCAAAAG‐3′, antisense 5′‐AATGCTACAGAAGGTGCTGGTGC‐3′, and the amplicon size was 138 base pairs (bp). Glyceraldehyde‐3‐phosphate dehydrogenase (GAPDH) was used as the internal control gene with the specific primers: sense 5′‐TCATGGGTGTGAACCATGAGAA‐3′, antisense 5′‐GGCATGGACTGTGGTCATGAG‐3′, and the amplicon size was 146 bp. The primers were synthesized by Shanghai Sangon Biological Engineering Technology & Services Co., Ltd. (Shanghai, China).

RNA and cDNA preparation and qRT‐PCR procedures were followed as listed in the literature. Briefly, total RNA was extracted from tissue samples using RNAiso Plus (TaKaRa Bio., Otsu, Japan) and quantitated using an ultraviolet spectrophotometer (Beckman Coulter, Brea, CA, USA) by measuring *A*_260_ and *A*_280_. The RNA of each sample was then reverse transcripted into cDNA using the PrimeScript^™^ RT Reagent Kit (TaKaRa Bio.) according to the manufacturer's instructions. Different sets of primers were tested for amplifying the targeted DNA from the cDNA by PCR by the *Premix Taq* ^™^ Kit (TaKaRa Bio.). The reactions were subjected to an initial denaturation at 95°C for 10 sec., followed by 40 cycles of denaturation at 95°C for 5 sec., and annealing and extension at 60°C for 45 sec. The PCR products were analysed on a 2% agarose gel and by sequencing (Shanghai Sangon) 20. Amplification efficacy of Arpin and GAPDH was examined by qRT‐PCR technique using SYBR *Premix Ex Taq* ^™^ II Kit (TaKaRa Bio.) in LightCycler 480 (Roche, Rotkreuz, Switzerland) using serial twofold dilutions of reverse‐transcribed cDNA. And only the amplification efficacies of the two genes were similar. The value of the threshold cycle (CT) calculation for the relative quantification of the target gene may be further quantified using the 2^−ΔΔCT^ method 21. The levels of Arpin and the control GAPDH mRNA transcripts were determined by qRT‐PCR and the value of the CT for each reaction was recorded. The levels of Arpin mRNA transcripts relative to GAPDH were expressed as ΔCT (CT_Arpin_ − CT_GAPDH_) and further quantified using the 2^−ΔΔCT^ method, where ΔΔCT = (CT_Arpin_ − CT_GAPDH_)_cancer tissue_ − (CT_Arpin_ − CT_GAPDH_)_normal tissue_ 22. Assays were performed in duplicate, and each experiment was repeated two times. Data were expressed as mean ± S.D.

### Statistical analysis

The association between the protein and clinicopathological parameters was analysed using the chi‐square test. The association between Arpin protein expression and axillary lymph node metastasis was assessed by univariate and multivariate logistic regression with covariate adjustment. Differences between groups were analysed using Student's *t*‐test or anova. Correlations between covariates were determined using Pearson correlation analysis. Survival curves for RFS were drawn by the Kaplan–Meier estimate, and comparisons between the survival curves were conducted using log‐rank tests. A Cox proportional hazards regression model was constructed to identify factors that were independently associated with RFS and to evaluate the independent impact of Arpin expression on RFS. Statistical analysis was performed using SPSS 16.0 software (SPSS, Chicago, IL, USA). All tests were two‐tailed, and *P* < 0.05 was considered to be statistically significant.

## Results

### Expression of Arpin protein is decreased in breast cancer tissues

In order to know the specificity of the Arpin antibody (sc‐242049; Santa Cruz Biotechnology Inc.), the antibody was first used to detect the endogenous expression of Arpin by Western blot assay (final dilution 1: 200) in paired tumour tissue samples, paratumour tissue samples and in breast normal cell line Hs578bst depleted or not of Arpin. The result showed that Arpin was significantly decreased in tumour samples compared with paratumoural normal tissues, and Arpin was inhibited greatly in Arpin‐depleted Hs578bst (Fig. S1). Although the endogenous Arpin protein is not very highly expressed in tissue samples, especially in cancer tissue samples, the results showed a clear major band at the right position of 25 kD. So, the Arpin antibody could recognize Arpin protein specifically.

**Figure 1:**
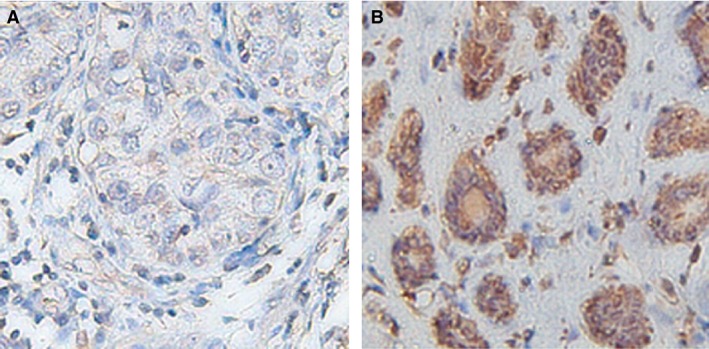
Representative photomicrographs of Arpin immunohistochemical staining. **A** indicates the high expression in normal breast tissue and **B** indicates low expression in breast carcinoma. Original magnification ×200.

From IHC assay, the results showed that the expression of Arpin protein was higher in normal tissues than that in cancer tissues (Fig. 1, Table 3). Whereas low and high expression could also be detected in a few normal breast tissues and breast cancer tissues, respectively, due to the heterogeneity of breast tumours (Fig. S2).

**Table 3:** IHC staining for Arpin in breast cancer and normal breast tissues

	No. of patients	Arpin expression		*P*
		High (*n* = 87)	Low (*n* = 132)	
Normal	43	31	12	0.001
Cancer	176	56	120	

### Expression of Arpin mRNA is decreased in breast cancer tissues

#### Primer specificity and amplification efficiency

To determine the levels of Arpin mRNA transcripts, total RNA was extracted from individual samples following reverse transcription into cDNA. As shown in the results, the DNA products were observed with Arpin (138 bp) and GAPDH (146 bp), respectively, suggesting the high specificity of the primers (Fig. S3). Further sequence analysis revealed that the PCR products were Arpin and GAPDH DNA fragments (Fig. S4). These preliminary results provided a reasonable basis for quantitative characterization of Arpin and GAPDH mRNA transcripts in the tissue samples. Amplification curves of serially diluted cDNA samples exhibited a standard S shape, suggesting good amplification efficiency and a linear relationship. For the Arpin gene, the log value of each cDNA dilution was plotted *versus* ΔCT, indicating that it had amplification efficiencies similar to GAPDH, justifying application of the 2^−ΔΔCT^ method for relative quantification 21.

#### Low levels of Arpin mRNA transcripts in breast cancer tissues

**Figure 2:**
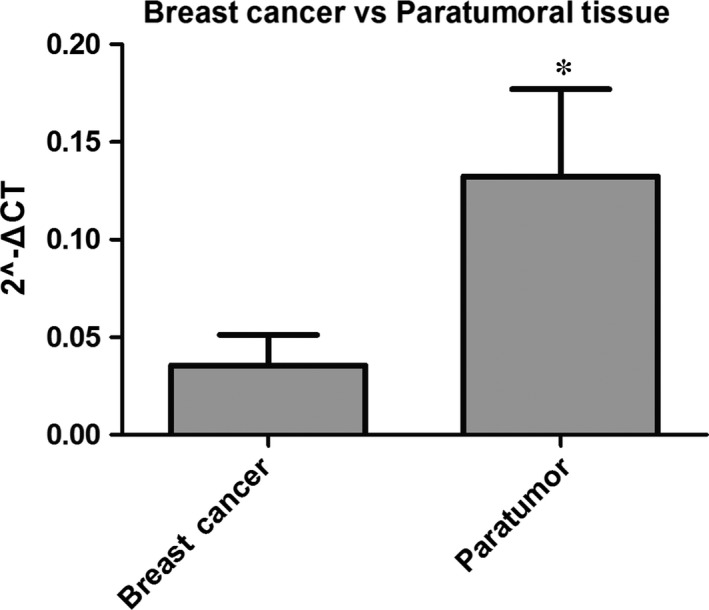
Relative expression levels of Arpin in breast cancer tissues and paratumoural tissue (*P < 0.05).

To acquire insights into the role of Arpin in breast tumourigenesis, 104 pairs of fresh breast cancer tissues and their matched paratumour breast tissues were used to assess Arpin mRNA expression using qRT‐PCR. The results showed that the Arpin mRNA expression level was significantly lower in breast cancer tissues compared with that of the matched paratumour tissues (*P* < 0.05). The median fold change in breast cancer tissues was only 0.27 of that in the matched paratumoural tissues (*P* < 0.05) (Fig. 2). So, the expression level of Arpin mRNA was significantly decreased in breast cancer tissues.

### The relationship between clinicopathological characteristics and Arpin expression

**Figure 3:**
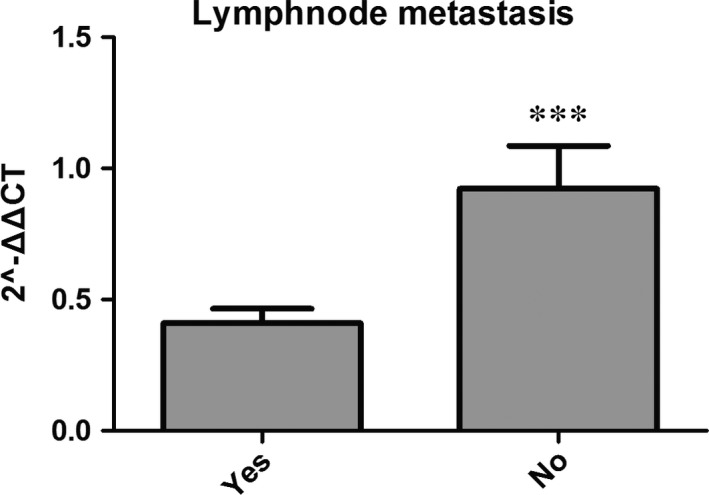
Relative expression levels of Arpin in LNM and LNM‐free breast cancers (***P < 0.05). LNM: lymph node metastasis.

Correlations between Arpin expression and clinicopathological characteristics are shown in Figure 3, Tables 1 and 2. In the 104 samples for qRT‐PCR, Arpin mRNA expression was significantly different between patients with and without lymph node metastasis (*P* < 0.05) (Fig. 3 and Table 2). Analysing the IHC results from 176 tumour samples showed that Arpin protein expression was lower in stage III than in stage I or II. Low Arpin protein expression was significantly associated with advanced TNM stage (III *versus* I, *P* < 0.05, Table 4). The chi‐square analysis of the 176 samples for IHC showed that the protein expression level of Arpin in tumour tissues was significantly correlated with lymph node metastasis (*P* < 0.05), PR (*P* < 0.05) and TNM stage (*P* < 0.05) (Table 1). Also, patients with positive PR expression or lymph node metastasis tended to show low Arpin expression. In contrast, there was no significant association between Arpin expression and other clinicopathological features such as patient age, tumour size, histologic type, expression of OR, Her‐2 and Ki‐67 as well.

**Table 4:** Association analysis of expression of Arpin protein *versus* TNM stage of breast cancer

TNM stage	No. of patients	Arpin expression		*P*
		High (*n* = 56)	Low (*n* = 120)	
I	72	32	40	0.138^a^
II	52	16	36	0.102^b^
III	52	8	44	0.001^c^

a Stage I *versus* II.

b Stage II *versus* III.

c Stage I *versus* III.

### Impact of Arpin low expression on axillary lymph node metastases in breast cancer

Among the 176 patients for IHC, 88 were free from lymph node metastasis and 40 showed high expression of Arpin. Of the 88 patients with axillary lymph node metastasis, 72 showed low expression of Arpin. The sensitivity and specificity of low‐expression Arpin for lymph node metastases were 81.8% (72/88) and 45.5% (40/88) respectively. Univariate and multivariate logistic regression with covariate adjustment were performed to evaluate the association between reduced Aprin expression and axillary lymph node metastasis. As the status of the axillary lymph node metastasis is included in the TNM stage, the TNM stage was not included in the analysis. We first performed univariate analysis of traditional clinicopathological variables and Arpin expression to identify the variables associated with axillary lymph node metastasis. The presence of lymph node metastasis was notably associated with tumour size (*P* < 0.05), PR status (*P* < 0.05), Her‐2 status (*P* < 0.05) and Arpin expression (*P* < 0.05). All variables significantly associated with axillary lymph node metastasis in the univariate analysis were included in a multivariate logistic regression model to find the best determinants or predictors of axillary lymph node metastasis. Results of the univariate and multivariate analyses are presented in Table 5. Low Arpin expression could be an independent predictor of axillary lymph node metastasis (Arpin: odds ratio: 3.242; 95% confidence interval: 1.526, 6.888, *P* < 0.05).

**Table 5:** Risk factors for 176 cancer patients with axillary lymph node metastasis (univariate and multivariate logistic regression analysis adjusted by age)

Variables	Univariate analysis			*P*	Multivariate analysis			*P*
	*B*	SE	OR (95% CI)		*B*	SE	OR (95% CI)	
Age (years)								
≥50	1.00 (reference)				1.00 (reference)			
<50	0.009	0.391	1.009 (0.469,2.172)	0.981	0.269	0.371	1.308 (0.633, 2.704)	0.469
Tumour size (cm)								
≥2	1.00 (reference)				1.00 (reference)			
<2	−1.544	0.470	0.213 (0.085, 0.536)	0.001	−1.205	0.419	0.300 (0.132, 0.682)	0.004
Grade								
1	1.00 (reference)							
2	−0.471	0.673	0.624 (0.167, 2.334)	0.484				
3	0.551	0.690	1.735 (0.449, 6.708)	0.425				
OR								
Positive	1.00 (reference)							
Negative	−0.025	0.720	0.975 (0.238, 3.999)	0.972				
PR								
Positive	1.00 (reference)				1.00 (reference)			
Negative	−1.248	0.632	0.287 (0.083, 0.991)	0.048	−1.447	0.484	0.235 (0.091, 0.607)	0.003
Her‐2								
Positive	1.00 (reference)				1.00 (reference)			
Negative	−3.613	0.818	0.027 (0.005, 0.134)	<0.001	−3.317	0.742	0.036 (0.008, 0.155)	<0.001
Ki‐67								
Positive	1.00 (reference)							
Negative	0.554	0.486	1.740 (0.671, 4.512)	0.255				
Arpin expression								
High	1.00 (reference)				1.00 (reference)			
Low	1.486	0.430	4.420 (1.094, 10.262)	0.001	1.176	0.385	3.242 (1.526, 6.888)	0.002

Ki‐67 ≤ 20%: Negative.

Ki‐67 > 20%: Positive.

OR: oestrogen receptor; PR: progesterone receptor.

### Correlation of Arpin expression and RFS in breast cancer patients

**Figure 4:**
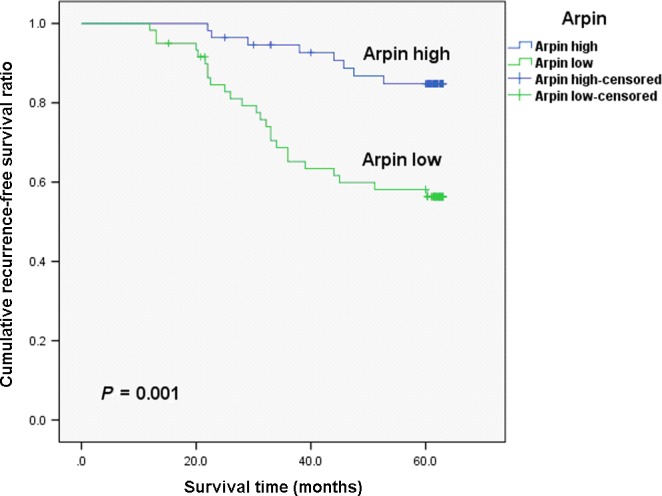
Kaplan–Meier curves of the recurrence‐free survival for breast cancer patients with high and low Arpin expression. *P*‐values were obtained by the log‐rank test.

To validate the clinical utility of Arpin expression, we evaluated the prognostic power of Arpin protein for 5 year RFS in 176 breast cancer specimens which were used for the IHC assay mentioned previously. The survival analysis revealed that patients with low Arpin expression showed a worse prognosis for RFS compared to those with high Arpin expression (Fig. 4, *P* < 0.05). To analyse the relevance of Arpin expression and clinicopathological features with RFS, a univariate Cox regression analysis was performed wherein factors associated with RFS included TNM stage (stage I and II *versus* stage III), lymph node status, tumour diameter, Arpin expression, OR status, PR status and HER‐2 status (Table 6). In addition to Arpin expression (*P* < 0.05), tumour diameter (*P* < 0.05), lymph node status (*P* < 0.05), and TNM stage (*P* < 0.05) significantly affected the RFS (Table 6). Multivariate Cox regression analysis indicated that low‐expression Arpin (*P* < 0.05) and tumour diameter (*P* < 0.05) were independent prognostic factors related to RFS.

**Table 6:** Univariate and multivariate survival analysis of RFS in 176 patients with breast cancer

Variable	Univariate analysis			Multivariate analysis		
	HR	95% CI	*P*‐value	HR	95% CI	*P*‐value
Age	0.112	0.668—1.875	0.669			
LN	2.984	1.694—5.256	<0.001	1.968	0.969—3.997	0.361
TNM stage (I + II *versus* III)	0.366	0.218—0.613	<0.001	1.011	0.509—2.010	0.974
OR expression	1.492	0.818—2.723	0.192			
PR expression	1.726	0.914—3.258	0.092			
HER‐2 expression	0.677	0.291—1.576	0.365			
Ki‐67 expression	0.728	0.420—1.259	0.256			
Diameter	3.769	2.241—6.339	<0.001	3.096	1.749—5.480	<0.001
Arpin	0.275	0.130—0.580	0.001	0.373	0.171—0.813	0.013

CI: confidence interval; RFS: recurrence‐free survival.

## Discussion

Metastatic diseases which have been reported to be controlled by specific genetic events contribute to the vast majority of cancer‐related deaths 23. The cascade of metastasis from the primary tumour to a distant site is a complicated process which contains acquisition of many intermediate phenotypes. The metastatic cascade involves a lot of changes in cancer cell movement, migration and invasion, and interaction with the extracellular environment 23, 24. To understand the mechanisms of every step is vital to help us to control metastatic disease and to design anti‐metastatic therapies.

Cancer cell migration is usually initiated by the formation of lamellipodia, the common process of which is driven by spatially and temporally regulated actin polymerization at the leading edge 6. Actin polymerization is regulated by a very important molecule—Arp2/3 complex—which is considered to be a key regulator of cell motility 7. Arp2/3 complex accomplishes its mission of nucleating actin filaments by binding actin filaments to the side of an existing filament and initiating branch formation 13. It is involved in the development and migration of some cancers such as pancreatic cancer, gastric cancer, colorectal cancer, breast cancer and so on 8, 9, 10, 11. Thus, the regulation mechanism of the Arp2/3 complex has been extensively studied and many factors were found to promote the function of the Arp2/3 complex 25. However, the negative regulation mechanism of the Arp2/3 complex is rarely studied and is still unclear.

Dang *et al*. 14 identified a new protein, Arpin, which was a negative regulator of the Arp2/3 complex. Arpin was utilized to modulate actin nucleation activity at the leading edge of the lamellipodium to steer the cell *via* inhibition of lamellipodia formation. Their experiments proved that Arpin indeed binds to the Arp2/3 complex and the levels of Arpin are inversely correlated with the speed and persistence of lamellipodia in the breast human cell line MDA‐MB‐231. However, the expression of Arpin in human breast cancer tissues is still unknown.

In order to investigate whether the level of Arpin is altered in breast cancer, we first compared the expression of Arpin protein in breast cancer tissues and normal tissues using the formalin‐fixed, paraffin‐embedded tissue from breast cancer surgical resection specimens. The results showed that Arpin protein was significantly less expressed in tumour samples than that in paired paratumoural normal tissues. The endogenous Arpin in breast normal cell line Hs578bst was inhibited greatly in Arpin‐depleted Hs578bst. So the Arpin antibody could recognize the endogenous Arpin protein specifically. Furthermore, to gain insights into the role of Arpin in breast tumourigenesis, we assessed Arpin mRNA expression in 104 pairs of breast cancer tissues and their matched paratumourous breast tissues using qRT‐PCR. The Arpin mRNA expression level was also significantly lower in breast cancer tissues compared with that of the matched paratumour tissues. So, the decreased Arpin level in breast cancer may lead to the acquisition of invasion and metastasis ability. We then accessed the correlation of Arpin expression and tumour staging. The results of IHC showed that low Arpin expression was significantly associated with advanced TNM stage. As TNM stage was defined by tumour size, regional lymph node and metastasis, we further analysed the correlation of Arpin expression and tumour size and the correlation of Arpin expression and regional lymph node. We found that there was no significant association between Arpin and tumour size. However, low Arpin expression was significantly associated with more axillary lymph node metastases. Such a result may explain that some axillary lymph node metastasis occurs when the tumour size is small.

More importantly, the present study found that the prognosis for RFS was significantly worse in breast cancer patients with low Arpin expression than in those with high Arpin expression. The recurrent cases of breast cancer generally include the regional recurrence and distant relapse. And the distant relapse cases usually occupy the majority of recurrent cases according to the rough statistics database of the Center of Diagnosis and Treatment of Breast Disease of our hospital. As reported previously, patients with lymph node involvement have a worse post‐relapse outcome and the number of lymph node metastases has significant correlation with the distant relapse 26, 27. Consistently, our study demonstrated that the expression of Arpin is an independent predictor of axillary lymph node metastasis and RFS. We presumed that Arpin probably played a role in breast cancer metastasis and such ability may suggest that breast cancer is apt to metastasis and occurrence of distant relapse. However, the exact molecular events leading to cancer metastasis have not yet been well elucidated and further research is still needed.

A limitation of our study is the limited number of cases and our findings should be further confirmed by large sample studies. As the number of death events in our enrolled cases is smaller compared to recurrent events, we only analysed the correlation between Arpin and RFS. Further studies are underway. To further clarify the role of Arpin as a prognostic biomarker in breast cancer, we will evaluate the association between Arpin expression and clinical outcome in a prospective cohort study.

There are several positive regulators of the Arp2/3 complex, such as Wiskott–Aldrich syndrome protein (WASP), neural WASP, WASP family verprolin‐homologous protein (WAVE; also known as SCAR) and WASP and SCAR homologue (WASH) complex 25. The interactions between Arpin and these proteins are still unclear. Further study is needed to determine the possible interactions between Arpin and positive regulators of the Arp2/3 complex.

## Conclusion

Our study provides evidence that Arpin expression is associated with the clinical characters and outcome in breast cancer patients. Its decrease predicts a poor outcome. Arpin may be an important biomarker for predicting unfavourable biological behaviour such as axillary lymph node metastasis and recurrence. To our knowledge, the present study is the first report conducted to determine the value of Arpin in patients with breast cancer, although the results need to be confirmed in a further follow‐up and with larger samples. Arpin may be used as a biomarker that could provide important tumour progress information and even a possible target for breast cancer therapy.

## Conflicts of interest

The authors confirm that they have no conflicts of interest.

## Supporting information


**Figure S1** Arpin expression in normal breast cells and in paired tumour and paratumoural normal tissues.

**Figure S2** Representative photomicrographs of Arpin immunohistochemical staining.

**Figure S3** Gel electrophoresis of PCR products. 1, 100‐600 bp markers; 2–3, PCR products of Arpin; 4–5, PCR products of GAPDH.

**Figure S4** Part of sequence chromatograms of Arpin and GAPDH PCR products.
