# Supplementary figures and images for: Aberrant expression of Arpin in human breast cancer and its clinical significance
Source: J Cell Mol Med. 2015 Dec 9;20(3):450–8. doi: 10.1111/jcmm.12740 (PMC4759471; doi:10.1111/jcmm.12740)

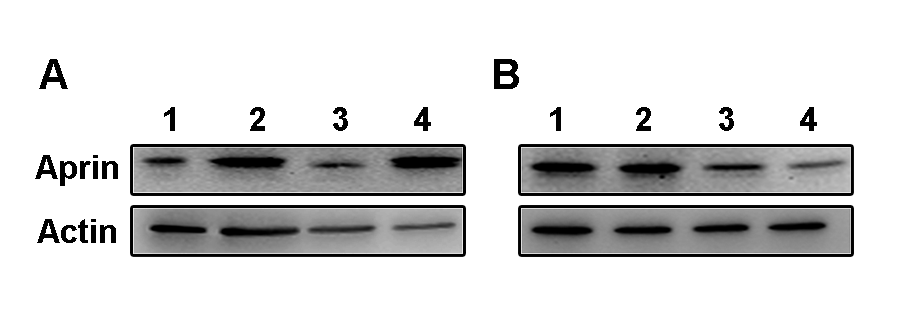

Supplement: Supplementary file 1 — Figure S1 Arpin expression in normal breast cells and in paired tumour and paratumoural normal tissues. [file JCMM-20-450-s001.tif]

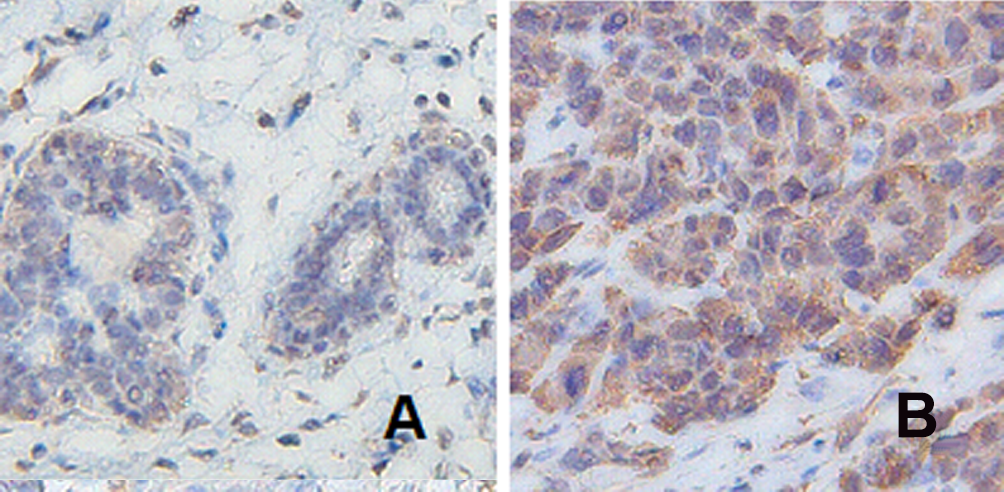

Supplement: Supplementary file 2 — Figure S2 Representative photomicrographs of Arpin immunohistochemical staining. [file JCMM-20-450-s002.tif]

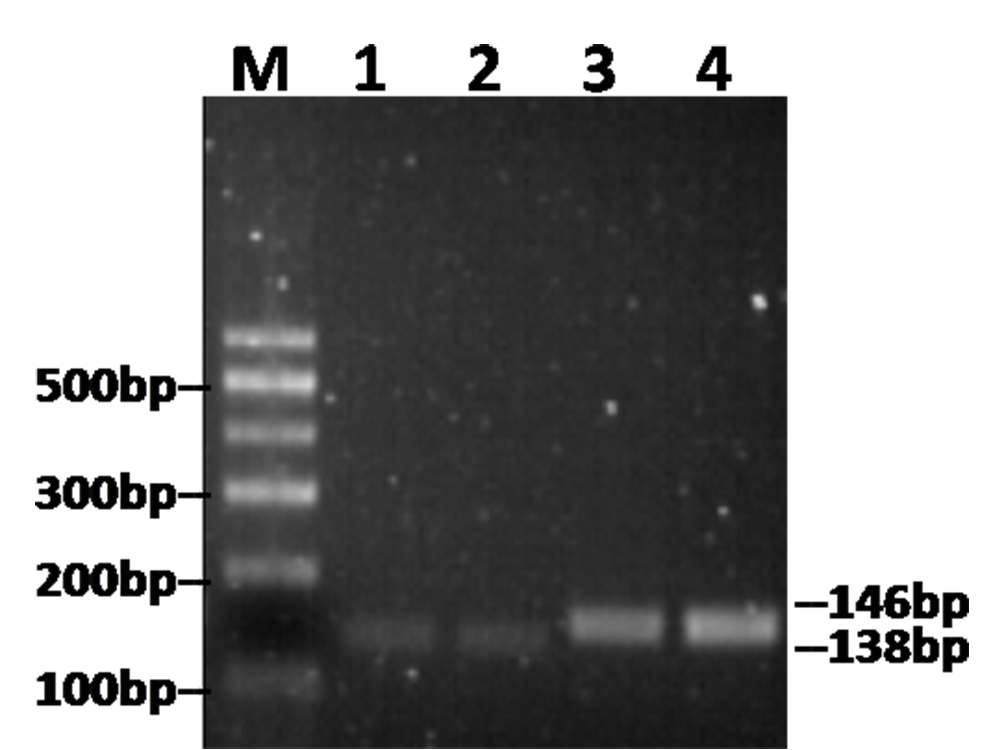

Supplement: Supplementary file 3 — Figure S3 Gel electrophoresis of PCR products. 1, 100‐600 bp markers; 2–3, PCR products of Arpin; 4–5, PCR products of GAPDH. [file JCMM-20-450-s003.tif]

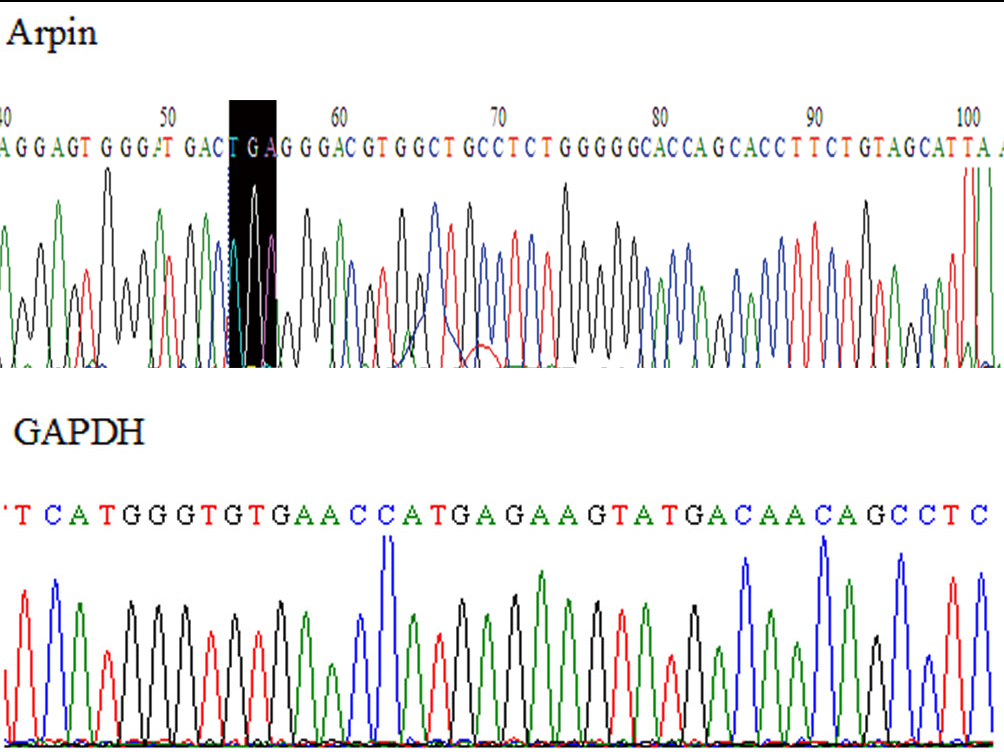

Supplement: Supplementary file 4 — Figure S4 Part of sequence chromatograms of Arpin and GAPDH PCR products. [file JCMM-20-450-s004.tif]
